# Microbial Pyrrolnitrin: Natural Metabolite with Immense Practical Utility

**DOI:** 10.3390/biom9090443

**Published:** 2019-09-03

**Authors:** Shraddha Pawar, Ambalal Chaudhari, Ratna Prabha, Renu Shukla, Dhananjaya P. Singh

**Affiliations:** 1 School of Life Sciences, Kavayitri Bahinabai Chaudhari North Maharashtra University, Jalgaon 425001, India (S.P.) (A.C.); 2 ICAR-National Bureau of Agriculturally Important Microorganisms, Maunath Bhanjan 275101, India (R.P.) (R.S.)

**Keywords:** Halometabolites, pyrrolnitrin, biosynthesis, biochemistry, spectral properties, antifungal activity, applications

## Abstract

Pyrrolnitrin (PRN) is a microbial pyrrole halometabolite of immense antimicrobial significance for agricultural, pharmaceutical and industrial implications. The compound and its derivatives have been isolated from rhizospheric fluorescent or non-fluorescent pseudomonads, *Serratia* and *Burkholderia*. They are known to confer biological control against a wide range of phytopathogenic fungi, and thus offer strong plant protection prospects against soil and seed-borne phytopathogenic diseases. Although chemical synthesis of PRN has been obtained using different steps, microbial production is still the most useful option for producing this metabolite. In many of the plant-associated isolates of *Serratia* and *Burkholderia*, production of PRN is dependent on the quorum-sensing regulation that usually involves N-acylhomoserine lactone (AHL) autoinducer signals. When applied on the organisms as antimicrobial agent, the molecule impedes synthesis of key biomolecules (DNA, RNA and protein), uncouples with oxidative phosphorylation, inhibits mitotic division and hampers several biological mechanisms. With its potential broad-spectrum activities, low phototoxicity, non-toxic nature and specificity for impacts on non-target organisms, the metabolite has emerged as a lead molecule of industrial importance, which has led to developing cost-effective methods for the biosynthesis of PRN using microbial fermentation. Quantum of work narrating focused research efforts in the emergence of this potential microbial metabolite is summarized here to present a consolidated, sequential and updated insight into the chemistry, biology and applicability of this natural molecule.

## 1. Introduction

Of 5–30 million species on the Earth, fewer than 2 million have been described and fewer than 1% have been explored for a vast repertoire of new natural products with socio-economic significance [1]. Hence, it is reasonable to expect that many more natural products not only from known species, but also from unidentified organisms are yet to come to benefit humanity and the environment [2]. Natural products offer unique structural molecules unparalleled by any other molecular family with an array of biological activities such as for drug leads. The many natural products that occupy the market today without any chemical modification are a testimony to the remarkable properties of secondary molecules produced by an array of plants, insects, animals, microbes and numerous species of marine organisms [3].

Secondary metabolites are small heterogenous organic molecules [4] that display prominent ecological benefits to the host organisms in providing defense against predators, parasites, diseases, interspecies nutritional competence, and competitive edge over interaction with the environment [5,6]. Extensive microbial structural diversification has led to maximizing chemical diversity in terms of the secondary metabolite resources that triggered scope for new drug leads [7]. Since natural products have reflected a wide array of therapeutic and biological applications (antibiotic, anti-inflammatory, antimicrobial, antitumor, anticancer, antiparasitic and immunosuppressing agents as well as enzyme inhibitors), the scope for further exploration of uncharacterized molecules of plant and microbial origin has always remained a focused area for identifying new leads for pharmaceutical and agro-chemical usages [8]. 

Continuously changing environmental patterns, the emergence of new diseases and resurgence of resistance towards existing drugs have led to an extensive search for novel natural metabolites at a rapid rate [9]. Low molecular size secondary metabolites from living entities have been obtained with typical therapeutic, biological and agricultural implications including antimicrobials, growth promoters, disease suppressers, enzyme inhibitors, health stimulators, and biocontrol agents against pathogenic fungi, bacteria and insects [10,11]. Systematic strategies for obtaining bioactive metabolites include isolation and identification of known secondary metabolites with biological activities unmatched with the molecular libraries or search for unknown natural molecules with versatile bioactivities. For both these options, the microbial world offers a great repository of natural molecules due to their extensive chemical diversity. However, there remains limitations of the culturability of microbial species and the expression of desired molecular traits or chemical species under isolated culture conditions. To overcome this, metagenomics has emerged to represent vast structural diversity of taxonomic communities with multi-functionalities having diverse chemical structures and functions [12]. 

Thus, secondary metabolites are supposed to be conserved in the species, evolved in a competitive environment, emerged to serve purposes other than primary metabolism, secreted for specific physiological or defense-related reasons, related with the habitat of producing organisms, blessed with complex chemical structures and clubbed with diverse bioactivity [13,14]. These attributes potentiate the usefulness of structurally diverse but functionally sound microbial biomolecules in therapeutics, and agricultural, industrial and environmental applications. We discuss structural, chemical, biological and functional perspectives of one of the earliest known pyrrole antibiotic antifungal metabolite of microbial origin, pyrrolnitrin, which has witnessed laboratory to commercial implications. 

## 2. Halometabolites with Potential Functions 

Secondary metabolites have emerged as a potential tool against many diseases after 1980 [15]. These molecules account for nearly 67% of the total antibiotics produced [16,17]. Secondary metabolites with halogen moiety in their chemical structure, referred to as “halometabolites”, display wide structural diversity with unique biological functions [18] (Table 1). Earlier, 29 halometabolites were reported with various functions [18] but now more than 5000 natural organohalogens, predominantly chlorinated and brominated compounds, have been identified [19]. These halometabolites are produced by several organisms including microbes, sponges, higher plants and insects. Organisms undergoing abiotic stresses such as extreme conditions, forest fires, volcanoes and volcanic eruptions that lead to abiotic oxidation of organic matter are more prone to halo-compound synthesis [20]. Initially, halometabolites were considered nothing more than an oddity, but later they attracted more attention because of their biogenesis, structural diversity and potential bioactivity.

In halometabolites, the halogen atom from halides ions (Cl−, Br−, I− and F−) is incorporated in organic compound with halogenation catalyzed by halogenase. Metabolites having bromine and iodine are mostly secreted by invertebrates and algae from marine habitats. Organisms from sea water habitat have comparatively more bromine content, while chlorinated metabolites were dominant in terrestrial species. Besides, fluorinated metabolites were also synthesized by few higher plants [30]. The 200-fold increase in the number of secondary metabolites with halo-molecules has been seen due to extensive research for antibiotics from marine habitats. It may have happened because incorporation of a halogen moiety potentiates more bioactivity and facilitates bioavailability of molecules [49]. Furthermore, the prevalence of halogen (Cl- or Br-) can offer a chemically reactive and orthogonal handle for selective modification through cross coupling chemistry [50]. The most common halogen found in secondary halometabolites is chlorine followed by bromine, while iodine and fluorine are considerably low [49,51]. Of these, chlorinated halometabolites has more advantages of being amenable to chemical modification for tailor-made bioactivity and increased drug efficacy [52]. The recent surge of interest in halometabolites seems to be due to their potentialities as effective alternative to current antifungal agents and, therefore, the pyrrolnitrin metabolite of soil microbial habitat holds promise. 

## 3. Pyrrolnitrin (PRN)

**Figure 1:**
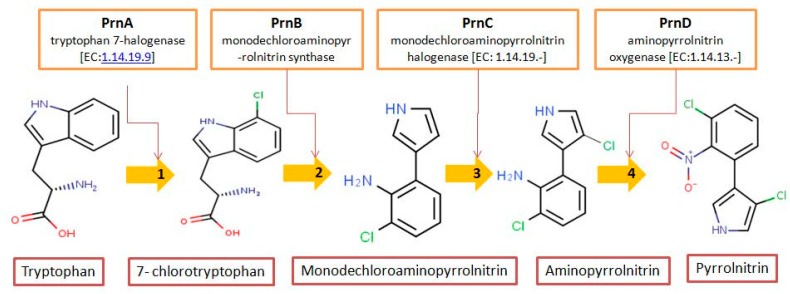
Biosynthetic steps in the synthesis of pyrrolnitrin. 7-chlorotryptophan is formed from tryptophan due to flavin-dependent halogenation catalyzed by the enzyme tryptophan 7-halogenase (PrnA). Further, the enzyme PrnB (monodechloroaminopyrrolnitrin synthase catalyzes formation of monodechloroaminopyrrolnitrin from 7-chlorotryptophan while the enzyme PrnC leads to catalytic reaction for the conversion of monodechloroaminopyrrolnitrin into aminopyrrolnitrin. In the last step, aminopyrrolnitrin is converted to pyrrolnitrin with the help of the enzyme PrnD (aminopyrrolnitrin oxygenase).

Pyrrolnitrin [3-chloro-4-(2-nitro-3-chlorophenyl) pyrrole] is a phenylpyrrole derivative containing two chlorine atoms and a nitro group. PRN, isolated from *Pseudomonas pyrrocinia* and various other pseudomonads, was classified as halometabolite in as early as 1964 [38]. Later, the compound was biosynthesized using tryptophan as supplement in the medium [53] and chemically synthesized by Nakano et al [54]. Biosynthesis of PRN in *Pseudomonas aureofaciens* ATCC 15926 has shown that L-tryptophan is a direct precursor (Figure 1) [53]. However, Hammil et al. [55] obtained high yield of PRN in D-tryptophan amended medium. Tryptophan analogs amended in the fermentation medium can also yield a series of PRN-like derivatives [56] (Table 2) with low antimicrobial activity than the native parent compound.

Structurally, PRN possesses benzene and pyrrole rings with chlorine atoms on both of them and nitro and chlorine units to form an unusual natural skeleton. It has chlorine moiety to contribute more towards biological activity [57] in comparison to its bromine derivative [58]. Consequently, several natural congeners of PRN such as amino-pyrrolnitrin, iso-pyrrolnitrin, 2-chloropyrrolnitrin, oxy-pyrrolnitrin, 4-fluoropyrrolnitrin, and 3-fluoro-3-dechloropyrrolnitrin have been reported. Brominated derivatives of PRN can be synthesized by replacing chlorine ion with bromine in the presence of sodium bromide. 

### 3.1. Pyrrolnitrin: Chemical Synthesis

PRN is positive towards Ehrlich’s reagent where pyrrole ring gets condensed with p-dimethylaminobenzaldehyde to form the violet color complex. Pauly’s coupling reaction yields red color [59] and gives a negative reaction to the ferric chloride nitro group detection test. PRN can be oxidized by chromic acid to form corresponding compound which on oxidation with permanganate, yields carboxylic acid [38]. 

Modern synthetic targets for chemical synthesis require regiospecific polysubstituted aromatic or heteroaromatic components [60]. PRN is chemically synthesized by α-block of pyrrole ring and 2-nitro-3-chloroacetophenone, and subsequent chlorination at 4-position and oxidation of methyles using sulfurilchloride followed by decarboxylation [54]. In another approach, 2-Methyl-4-(2-nitro-3-chloro-phenyl)-5-ethoxycarbonyl-pyrrole was prepared in various steps from 2-amino-3-chloro-toluene [61]. One of the most versatile synthetic approach for PRN allowed access to analog compounds such as monodechloroaminopyrrolnitrin and aminopyrrolnitrin. This step facilitated PRN synthesis using Suzuki–Miyaura cross-coupling of an appropriately halogenated pyrrole pinacolboronate ester with halogenated arylpyrroles using 2,6-disubstituted nitrobenzenes or 2,6-disubstituted anilines [62]. Palladium-catalyzed coupling of 1-(triisopropylsilyl-3-substituted pyrroles with arylhaildes has also been described [63]. However, chemical synthesis of PRN makes synthetic route cost extensive and pose threat to the environment [62]. Furthermore, the chemical process utilizes noxious chemicals, high temperature and pressure, more energy and yield poor regioselectivity with lack of public acceptability [49]. Thus, chemical industries prefer microbial species for more selective, greener and cost-effective approach for synthesizing PRN.

### 3.2. Microbial Pyrrolnitrin Production and Recovery

Microbial synthesis of PRN is easy, reliable and eco-friendly and requires low-cost medium constituents, ambient conditions for growth and production, the least additional energy requirements and minimum expensive equipment. This is the major reason microbial synthesis of PRN has become the preferred alternative to chemical processes [49]. After initial isolation of PRN from *Pseudomonas pyrrocinia* [38] and thereafter reports from different fluorescent and non-fluorescent *Pseudomonas* species [53,64], several strains of *Burkholderia cepacia*, *Corallococcus exiguus, Cystobacter ferrugineus, Enterobacter agglomerans, Myxococcus fulvus, Serratia* spp. and *Actinosporangium vitaminophilum* have been classified to produce PRN in varying quantities [65,66,67,68]. *Serratia plymuthica* [69] and *S. ruhidaea* [70] are identified for enhanced production of PRN. Recently, a strain belonging to *Burkholderia cepacia* complex, JKB9, showing broad-spectrum antifungal activity, was held responsible for suppressing growth of *Phytophthora capsici*, *Fusarium oxysporum* and *Rhizoctonia solani* [71]. This strain, which has shown stronger antifungal activity than *Burkholderia* strains KCTC2973 and ATCC25416 against *Phytophthora* blight, was confirmed for PRN production using thin layer chromatography (TPC), high performance liquid chromatography (HPLC) and Nuclear Magnetic Resonance (NMR) spectrometric studies. Complete genome sequencing of *Burkholderia pyrrocinia* 2327^T^ revealed insights into the cells possessing antibiotic capabilities for the biosynthesis of PRN [72]. Cloning of gene clusters responsible for encoding enzymes involved in the production of pyrrolnitrin in organisms has greatly helped in marking of the biosynthetic routes. Using an antibiotic producing strain of *P. fluorescens* [73] cloned four gene clusters to elucidate biochemistry of these molecules and to link it with the enzymes that may offer the routes for the synthesis of new chemical structures. Earlier, *prnABCD* operon from *P. protegens* Pf-5 was co-expressed in tomato plants with universal vector IL-60 and successfully demonstrated resistance to damping-off disease caused by *R. solani* [74].

Microbial wild type strains secrete PRN in low quantity (Table 3) and production varies with the medium constituents. *P. aureofaciens* ATCC 15926 strain when grown in minimal medium, secreted PRN in low concentration (<0.3 µg mL^−1^). Even optimized variation of constituents in growth medium could not increase PRN production. However, the production enhanced by 30-fold when *P. aureofaciens* ATCC 15926 was mutated with N-methyl-N’-nitro-N-nitrosoguanidine [75]. Addition of DL-tryptophan (1 mg mL^−1^) in CMM medium also doubled PRN production after 120 h but additional amount of tryptophan resulted in less yield [76].

Besides intracellular production of PRN from *Pseudomonas* spp., the excretion of the compound was also detected in the supernatant of fermented medium of *Serratia marcescens* strain ETR17 [85]. B. cepacia yielded 0.54 mg L^−1^ of PRN in monosodium glutamate medium at 27 °C as quantified by preparative HPLC [66]. Initially, Elander et al. [64] reported that only 27.58% *Pseudomonas* spp. secreted PRN in shake flask fermentation propagated in CMM, C, or E media. The authors concluded that *P. multivorans* C653 (ATCC 17760) showed maximum PRN production in medium C, followed by E and then CMM. *P. aureofaciens* was shown to secrete moderate PRN in CMM medium (40–80 µg mL^−1^). The PRN concentration increased in D-tryptophan amended medium where it was incorporated in the biosynthesis of PRN.

While growing *P. aureofaciens* in isotopically labeled tryptophan (at different positions) containing medium, Martin et al. [86] demonstrated that amino nitrogen of D-tryptophan became the nitro group of PRN. The two chlorine atoms in PRN, C3 of side chain became pyrrole and C2 of the indole nucleus got retained during biosynthesis (Figure 1). Furthermore, Chang et al. [77] confirmed that H-2 and H-α of the indole and side chain give rise to H-5 and H-2 of PRN, respectively, and, thus, proposed that L-tryptophan is the immediate precursor in PRN biosynthetic pathway. PRN formation using labeled tryptophan showed that L- rather than D-tryptophan was the immediate precursor of PRN [87]. 7-chloroindole-3-acetic acid, 3-chloroanthranilate detected in fermented medium revealed that 7-chlorotryptophan served as a common precursor for PRN [88].

Variety of production media and their pH remained a key parameter to influence PRN secretion. Shake flask fermentation of *P. cepacia* LT4-12-W revealed that the final yield (at 168 h) of PRN almost doubled at pH 5.8. Amendment of MS medium with glutamate salt of sodium yielded 60.50 mg mL^−1^ of PRM secretion [89]. The effect of different physicochemical conditions on plasmid-mediated PRN secretion has also been reported from *Acinetobacter haemolyticus* A19 isolate from wheat rhizosphere [83]. 

Recovery strategy of PRN involves cell growth in appropriate medium, extraction in acetone followed by removal of oily matter from concentrated acetone solution using petroleum benzene [38]. From fermented broth at pH 10 or 11 (6 mL) with NaOH, cell pellet centrifugation following sonication with acetone (600 µL) for 1 min, separation of acetone supernatant, re-extraction of pellets again in acetone (300 µL) and drying of acetone extract also yield PRN extract [59]. Further, fermented cultures were extracted after 48 h with equal volume of ethyl acetate [90] and centrifuged. Pellets sonicated twice with ethyl acetate (5 mL) for 3 min then recovery of organic phase [91] resulted in PRN rich dried extract [92]. Majumdar et al. [83] reported lysis of 18 h culture of *Acinetobacter haemolyticus* A19 using 1% SDS followed by sonication for 5–15 min and supernatant collection for PRN. In the case of bioactivity and characterization study, chromatographic separation techniques such as column chromatography and flash column with different mobile phases were explored (Table 4). 

### 3.3. Analytical Characteristics of Pyrrolnitrin

PRN is chemically substituted with 3-phenyl pyrrole derivative containing two chlorine atoms and a nitro group [57]. The compound is a pale-yellow crystal [38], 3-chloro-4-(3-chloro-2-nitrophenyl)-1H-pyrrole, a phenylpyrrole molecule having a chemical formula of C_10_H_6_O_2_N_2_Cl_2_ and molecular weight 257.07 gmol^−1^. The melting point of PRN, which is sparingly soluble in water, petroleum ether and cyclohexane, but more soluble in ethanol, butanol, ethyl acetate, ethyl ether, snf chloroform, is 124.5 °C. Elemental analysis of the compound reflected C, 46.71%; H, 2.33%; O, 12.45%; N, 10.89%; and Cl, 27.68% [38]. PRN separation was achieved by different methods like chromatography TLC and HPLC [65,82] while structural features have been elucidated using Fourier transform infrared spectroscopy (FTIR) [93], nuclear magnetic spectroscopy (NMR ^1^H and ^13^C) [89] and mass spectroscopy (LC-MS and GC-MS) [99]. 

Separation of PRN from bacterial media extract using TLC utilized various stationary phases such as silica gel G, GF_254_, 60 F_254_, KCI8 F, C18 Glass and several mobile phases. PRN can be detected on TLC under UV transilluminator [83,100] and visualized by spraying diazotized sulfanilic acid (DSA) or Pauly’s, Ehrlich’s and van Urk’s reagent to develop maroon and violet color, respectively [101,102] or H_2_SO_4_ on Silica Gel G plate [64]. The R*_f_* value for various TLC system served to identify PRN from different bacterial species. The compound has been analyzed by retention time in gradient HPLC system [65] but isocratic solvent system of 45% water, 30% acetonitrile, and 25% methanol also separated pyrrolnitrin at 252 nm in preparative HPLC [102]. Modifications in the polarity of solvents, mobile-stationary phase and elution methods are key strategies to quantify PRN using HPLC (Table 5). Yellow colored PRN molecule isolated from *Pseudomonas pyrrocinia* absorbs at 252 nm with molar extinction coefficient of ε = 7500 in ethanol [26]. Myxobacterial PRN also showed λ_max_ at 252 nm in methanol [94]. Functional group stretching in FTIR vary with different PRN derivatives due to its structural features. Typical bond stretching at 1530 and 1375 cm^−1^ characterized for nitro group [38] while 3489 cm^−1^ represent pyrrole ring. Similarly, PRN isolated from supernatant of fermented medium inoculated by *Myxococcus fulvus* strain Mx f147 indicated infrared spectrum to confirm pyrrole ring (3460), nitro group (1530 and 1375), CH_3_ (stretch) (1460) and C=C aromatic weak intensity (1600) [94]. Mass spectroscopy (MS) of PRN is ascertain using different ionization techniques. MS of PRN isolated from *Pseudomonas cepacia* B37w showed molecular ion at *m/z* 256 with the formula C_10_H_6_C_12_N_2_O_2_ [59]. Electrospray mass spectroscopy (negative ion spectrum) of PRN further confirmed (mass-to-charge ratio; *m/z*) at 256 [66]. High-resolution mass spectrometry of the two molecular ions gave *m/z* 255.9826 and 257.9777, respectively, indicating the molecular formula C_10_H_6_N_2_O_2_^35^C_12_ and C_10_H_6_N_2_O_2_^35^Cl^37^Cl [99].

NMR spectroscopy is widely used for analytical measurement of microbial metabolites. The PRN is confirmed by NMR spectrum [59] with values: (i) 1H NMR: H-2 and H-5: 6.82 (m, 2H); H-5′: 7.41-7.53 (m, 3H); NH: 8.29 (br s, 1H); and (ii) 13C NMR δ value: 111.9 indicated C-3, 115.4 for C-4, 116.6 for C-5, 117.4 meant for C-2, while 124.8, 127.6, 128.6, 130.1, and 130.3 designated for C-3′, C-1′, C-6′, C-4′ and C-5′, respectively. Chemical shift (δ) values at 6.81 (m, 2H) indicate the presence of H-2 and H-5, 7.41: H-6′, 7.43: H-5′, 7.52: H-4′, 8.38: NH [89]. NMR spectrum of purified PRN secreted by plasmid-mediated *A. haemolyticus* A19 revealed the values δ: 6.2–6.6 (m, 2H, H-2, H-5), 6.77 (q 1H, H6), 7.03 (m, 1H, H-4), 7.38 (m2H, Ha, Hc) compared with standard 1H NMR spectrum of [65]. PRN synthesized from *Myxococcus fulvus* strain Mx f147 showed 13C NMR spectrum (in acetone-*d*6; Bruker 400 MHz) [94]. Structural investigation of PRN with X-ray analysis revealed the presence of two molecules with observed density of 1.74 g/cm^3^ that lie opposite to each other about the center of symmetry. It further confirmed the location of two Cl atoms in the asymmetric unit with 3D Patterson function, dihedral angle of the pyrrole, the benzene rings and chlorine substitution on pyrrole ring located apart from the nitro group [109].

### 3.4. Biochemistry of Pyrrolnitrin

Microbial synthesis of PRN requires D-tryptophan, but cost of precursor amino acid and intracellular secretion limits its large-scale production. The NO_2_ group is derived from anthranilic acid, phenylalanine and tryptophan that could serve as a precursor for PRN secretion [57]. However, anthranilic acid and L-phenylalanine usually decrease PRN secretion in *P. aureofaciens* and *B. cepacian* [66], while tryptophan stimulates PRN production [57,101]. In the medium, L-tryptophan gets quick intake within the cells than the D- isomer but addition of L-isomer could not yield more PRN secretion [110]. In actinomycetes, D-tryptophan enhances secretion of PRN when added separately in the culture medium [101] and maximum accumulation was observed at stationary phase after 120 h [66,101]. It indicated that the L-isomer of tryptophan enter cells quickly and participate in the protein synthesis, while D-tryptophan enter slowly and available at the time of antibiotic secretion [55]. Besides, L-glutamic acid amended medium showed maximum antifungal activity, which substantially declined with the addition of L-tryptophan, L-valine, L-serine, L-phenylalanine and L-cysteine [66]. In brief, D-tryptophan and L-glutamic acid are more direct precursors of PRN than any other amino acids.

**Figure 2:**
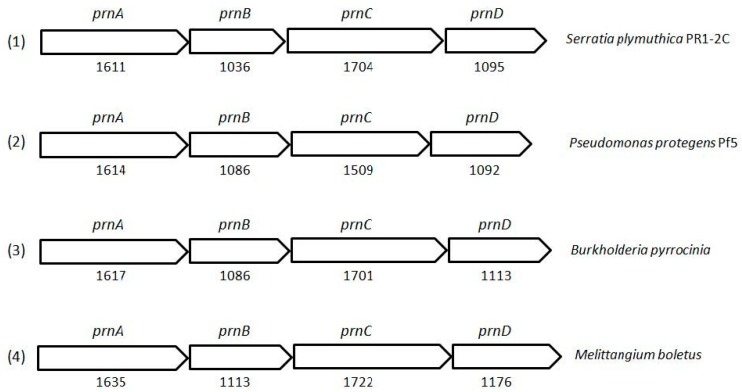
Cluster organization of pyrrolnitrin biosynthetic genes in *Serratia plymuthica* PR1-2C (1), *Pseudomonas protegens* Pf5 (2), *Burkholderia pyrrocinia* (3) and myxobacterium *Melittangium boletus* (4). Nucleotide sequence (NT size indicated for *prnA–D* genes) of the bacterial species was obtained from KEGG database.

PRN biosynthesis was unraveled in *P. aureofaciens* [77,101]. Later, genes (*prnABCD* operon) and corresponding enzymes involved were delineated in *P. fluorescens* BL915 (Figure 2) [90,111]. The biosynthesis of PRN occurs in four sequential steps: chlorination by prnA, rearrangement and decarboxylation by prnB, chlorination by prnC and oxidation by prnD enzyme (Figure 1). This involves regioselective halogenation of tryptophan through the addition of chlorine into D-tryptophan by tryptophan 7-halogenase (prnA) following nucleophilic and electrophilic reactions [112] and activation of intermediate lysine-chloramine species as the first step [113,114]. Further, the reaction catalyzed by prnB shows structural similarity with two-domain indoleamine 2,3-dioxygenase enzyme (IDO) and involves several intermediary steps. The second step forms a binary complex that combines with L-tryptophan or 7-Cl-L-tryptophan to create a ternary complex. The third step in PRN biosynthetic pathway of *P. fluorescens* leads to catalytic conversion of mono-chloro-deamino-pyrrolnitrin into amino-pyrrolnitrin by regioselectivity using halogenating and chlorinating enzyme [115]. In the last step, prnD catalyzes the oxidation of amino group of aminopyrrolnitrin to nitro group and thus forms PRN [90,111,116].

Aminopyrrolnitrin oxidase or arylamine oxygenase (rieske N-oxygenase) catalyzes oxidation of an arylamine into the arylnitro group. Except prnB, trptophan-7-halogenase (prnA), monodechloroaminopyrrolnitrin (prnC) and aminopyrrolnitrin oxidase (prnD) enzymes require flavin reductase (*prnF*) gene located close to the *prnABCD* operon which is considered as a part of the cluster [117]. Bioinformatics clubbed with the biochemical tools identified the role of *prnF* gene in *prnD*-catalyzed unusual arylamine oxidation in *Pseudomonas fluorescens* Pf-5 [118]. The *prnF* and *prnD* genes form a two-component oxygenase system, in which the gene product enzyme prnF supplies the reduced flavin to prnD. The prnF requires NADH as an electron donor to reduce FAD so that reduced FAD supplies electrons from NADPH to the prnD oxygenase component through protein-protein interactions in order to protect the flavin from oxidation.

The *prnF* gene having molecular mass of 17kD with GC content of 62%, encodes for a polypeptide chain of 160 amino acids. The enzyme belongs to flavin:NAD(P)H reductases family with part of two-component monooxygenase systems and its C-terminal region possesses highly conserved GDH motif for NAD(P)H binding [119]. It resembles with PheA2, SnaC, VlmR, ActVB and HpaC with 31.5%, 28.6%, 26.4%, 25.6%, and 25.5% amino acid identity, respectively [120,121,122,123,124,125,126].

### 3.5. Pyrrolnitrin Derivatives

Several halogen variations of the PRN molecule have been isolated in the past in the form of bromo-analogs of pyrrolnitrin from fermentation of *Pseudomonas aureofaciens* in sodium bromide with low antifungal activity [58]. In addition, 2-chloropyrrolnitrin contain an additional chlorine atom which possesses about 10% of the antifungal activity of PRN [55]. The pyrrolomycin (B, C, D, E, F_1_, F_2a_, F_2b_, and F_3_) derivatives encompass a chlorine or bromine atom at the 3-position of the pyrrole ring, and either two chlorine atoms at positions 4 and 5 or one chlorine and one bromine at any of these positions have shown significant antifungal activity [57]. Novel oxidized derivatives of pyrrolnitrin including two new pyrrolnitrin analogs, namely 3-chloro-4-(3-chloro-2-nitrophenyl)-5-methoxy-3-pyrrolin-2-oneand4-chloro-3-(3-chloro-2-nitrophenyl)-5-methoxy-3-pyrrolin-2-onehave, were reported from *B. cepacia* K87 [97].

Furthermore, number of *de*-chloro and *de*-nitro derivatives of PRN and the isomers were synthesized by cyclization of enamine reaction, hydrolysis, carboxylation and Mannich’s reaction [127]. The strongest antifungal activity of PRN and its analogs resulted when it got unsubstituted by ester group at any position. The antifungal activity become more stronger when shift of NO_2_ group was increased. Few PRN derivatives such as denitropyrrolnitrin (3-chloro-4-(3-chlorophenyl), bromo analog: 3-chloro-4-(3-bromophenyl)pyrrole) and trifluoromethyl derivative (3-chloro-4-(3-trifiuorornethyl) pyrrole) were strong antimicrobials. However, among all the analogs homologous to NO_2_ group of pyrrolnitrin, PRN has remained the strongest biologically active compound. The UV irradiation of 2-(pyrrol-3-yl)nitrobenzene moiety of PRN in an anhydrous aprotic solvent yielded 7,4′-dichlorospiro(1,3-dihydrobenzo(c)isoxazole-3,3′-pyrrolin-2′-one) by the intramolecular oxidation. Hence, the photodegradation of PRN depends on aqueous reaction media and the nature of its excited state [98].

## 4. Applications of Pyrrolnitrin

### 4.1. Biological Activity

Structure–activity mechanism reveals that the primary target of PRN lies in the cell membrane to impede protein, RNA, DNA synthesis and uncouple the normal electron flow in the respiratory electron transport chain [128]. The metabolite has demonstrated biological activity at low concentration and act as an uncoupler of oxidative phosphorylation in *Neurospora crassa*. High concentration of PRN causes impairment of electron transport in flavin region and cytochrome c oxidase; accumulation of glycerol; synthesis of triacyl glycerol leading to leakage of cell membrane and inhibition of cell growth; in vitro activity against bacteria and fungi in the range of 1–100 µg mL^−1^; in vitro activity against leukemia and melanoma cell lines; and moderate antimycobacterial activity at 8 µg mL^−1^ [129]. The halometabolite was used as a drug lead for fenipoclonil and fludioxonil synthesis [130]. The amino derivative of PRN was identified as an androgen receptor antagonist [131]. PRN has the unique property to persist actively in the soil over a month, and can be readily diffused and slowly released after lysis of host bacterial cell [132]. However, the compound is sensitive to decomposition due to light [98].

Inhibitory effect of PRN is seen on the mitochondrial electron transport system of *Neurospora crassa* 74A [66]. Studies using N,N,N’,N’-tetramethyl-p-phenylenediamine dihydrochloride (TMPD) confirmed that PRN block transfer of electron between the dehydrogenases and cytochrome c-oxidase components of the respiratory chain. At low concentrations, PRN uncouples oxidative phosphorylation in *Neurospora* mitochondria and impedes electron transport in both the Flavin region and cytochrome C oxidase at high concentration [133]. PRN also function as a signal molecule, beyond its role as a bioactive molecule to suppress fungal and affected cell motility [134]. Antifungal activity of the compound increased at pH 6.0, became maximum at pH 10 or 11 and declined after pH 11. Temperature influence on antifungal activity was maximum at 28 °C. Similarly, 2% NaCl content in the medium showed maximum activity. Such studies indicated more scope for medium modifications for obtaining maximum PRN production followed by maximizing biological activity of the compound.

The quorum-sensing system related regulation of PRN is reported in a chitinolytic bacterium *Serratia plymuthica* strain HRO-C48, that protects oilseed rape crop from *Verticillium wilt* [102]. The mutant deficient in PRN production shown the ability to produce the compound in the medium supplemented with chemically synthesized N-hexanoyl-HSL and N-3-oxo-hexanoyl-HSL (OHHL) (100 µM), thus suggesting that quorum sensing (QS) ability regulated PRN biosynthesis. While investigating the role of N-acylhomoserine lactone (AHL)-dependent quorum sensing for expressing antifungal traits, Schmidt et al. [135] found that PRN expression was positively regulated by *CepR* gene at transcription level. PRN is reported to have significant antimicrobial potential (Table 6) against *Streptomyces antibioticus, S. violaceoruber, Paecilomyces variotii* and *Penicillium puberulum*. However, *S. prasinus*, *S. ramulosus, Aspergillus proliferans* and *A. terreus* showed tolerance to pyrrolnitrin. PRN displayed activity against *Ustilago maydis*, *Candida albicans*, *Hansenula anomala*, *Arthrobacter oxidans*, *Bacillus coagulans, B. lichenifernis, B. subtilis* and *B. thuriengiensis* at low concentrations [66]. Pyrrolnitrin produced by *Pseudomonas chlororaphis* strain PA23 exhibited nematicidal and repellent activity against *Caenorhabditis elegans* [84]. Co-culturing *P. chlororaphis* and *C. elegans* enhanced expression of biocontrol-related *phzA, hcnA, phzR, phzl, rpoS* and *gacS* genes and, thus, contributed to the fast killing of nematode in bacterial interaction.

Bacterial growth inhibition by PRN forms complex with phospholipids of cell membranes that eventually cease cellular respiration [138]. Furthermore, PRN causes leakage of A260 mµ absorbing material inside the cells and impairs synthesis of protein, DNA and RNA [138]. However, in vitro protein synthesis in PRN treated *Rhizoctonia solani* and *Escherichia coli* remained unaffected [139]. It bursts protoplast of *Bacillus megaterium* KM at growth inhibitory concentration [138]. The multitudes and range of activity of PRN makes it a preferred bioactive compound for agricultural chemical sector.

### 4.2. Agricultural Applications

Phenylpyrroles were proven and effective agents against *Trichophyton, Microsporium, Epidermophyton, Penicillium, Candida spp*. and several Gram-positive bacteria [57]. Besides, PRN showed activity against soilborne fungal phytopathogens *Rhizoctonia solani* [140] and *Fusarium sambucinum* [33] and foliar fungal pathogens *Fusarium graminearum, F. culmorum* [141], *Pyrenophora triticirepentis* [142], *Thielaviopsis brasicola, Verticillium dahlia* and *Alternaria spp* [143]. The compound inhibited *Gaeumannomyces graminis* of wheat, *Alternaria brassicae* and *Botrytis cinereal*, and partially inhibited *Fusarium roseum*. Remarkable inhibition of mycelial growth and conidial germination was observed at a PRN concentration of 0.4 µg mL^−1^ compared to phenazine-1-carboxylic acid at 50 μg mL^−1^ [144]. Results suggest strong possibility of the compound being a prospective biocontrol agent in the agriculture.

The fungistatic effect of PRN was most distinct against Alternaria sp., Botrytis cinerea, Pythium aphanidermatum, P. ultimum, Rhizoctonia solani, Rhizopus sp. Aspergillus niger, Fusarium oxysporum, Penicillium expansum, and Sclerotium rolfsii [65]. Antibacterial activity was also recorded against Agrobacterium tumefaciens, Corynebacterium insidiousum, Pseudomonas syringae pathovar syringae, and Xanthomonas campestris (Minimum Inhibitory Concentration (MIC) ≥1 μg mL^−1^). Organisms such as Clavibacterium michiganense and Serratia marcescens were suppressed at MIC ≥ 10 μg mL^−1^ [65]. Moderate activity against Gram-positive and Gram-negative bacteria was seen at 12.5–100 mg mL^−1^ (MIC). Strong toxicity was noticed against fungi, especially trichophytes, Trichophyton asteroids, at MIC of 0.05 mg mL^−1^. In addition, PRN as a nitro-heterocyclic chemotherapeutic agent exhibited antimycobacterial activity against M. tuberculosis and M. avium [129]. At present, only two synthesized derivatives of PRN, namely fludioxonil and fenpiclonil analogs, were registered as agricultural fungicides in France and Switzerland, respectively. Commercial products of fenpiclonil and fludioxonil include BERET, GALBAS and SAPHIRE, CELEST and MAXIM sold by Syngenta, respectively [145].

PRN found most prolific applications in controlling damping-off disease of cotton and cucumber, tan spot of wheat, storage molds of pome fruits, seedling disease of cotton, dry rot of potato and sclerotinia wilt of sunflower [73]. More usage of the compound lies in its significant antibiotic activity and low toxicity to mammalian species [146]. Wounds on apple and pear were challenged with a conidial suspension of antagonist grey mold *B. cinerea* and blue mold *Penicillium expansum* to investigate the efficacy of pyrrolnitrin (6–200 µg mL^−1^) to control diseases at 2 and 24 °C after harvest. High concentrations of PRN proved effective at 24 °C on both diseases of apple and pear, while low concentrations appeared effective at cold temperature [147]. Hence, PRN is an attractive strategy to control postharvest diseases on fruits, vegetables and other agricultural products being produced at low temperature conditions. In a preliminary field experiment on strawberries, postharvest treatment with PRN (250 mg L^−1^) at low storage temperature delayed development of post-harvest rot by 2–4 days, but did not reduce rate of development [79] and spoilage to acceptable levels.

In greenhouse studies, PRN showed prominent activity against *Pyricularia oryzae* and *Botrytis cinerea* [148]. The PRN producer *P. chlororaphis* O6 has shown antifungal activities both in vitro and in planta [82] on tomato against late blight disease and demonstrated major antagonism. In addition, biocontrol of fungal disease *Fusarium* Head Blight (FHB) caused by *F. graminearum* on wheat heads in growth chamber conditions was studied using strain *Pseudomonas chlororaphis* G05 co-treated with: (i) wild-type strain G05; (ii) *phz*-deleted mutant G05Δ*phz*; and (iii) mutant G05Δ*prn.* The experiment showed wheat heads were infected with *F. graminearum* at rates of 5–8% and 80–90%, respectively, when co-sprayed with wild-type strain G05 and mutant G05Δ*prn* [144], and PRN of wild type strain was found to be vigorously active against FHB disease.

The glasshouse experiments with talc-based formulation of *S. marcescens* ETR17 were similar to in vitro studies. Incidence of root rot in bacteria treated tea plants were considerably lower in comparison to untreated control as well as the fungicide treated sets. Additionally, ETR17 formulation also increased the root and shoot length of the tea seedlings under both sterile and unsterile soil conditions in comparison to the untreated controls [85].

### 4.3. Pharmaceutical Applications 

Pyrrolnitrin demonstrated strong protecting activity against various pathogenic fungi, especially against dermatophytosis [149]. It has been recommended for the treatment of superficial fungal infection of dermatophytic *Trychophyton* in Japan [150,151]. A patent has been granted on antifungal composition containing pyrrolnitrin and antimycotic imidazole compound in 1987 [152]. The product was commercialized under trade name Pyro-Ace W powder Spray by Fujisawa Pharmaceutical Company Ltd., Osaka. This was marketed by Pharmacia in Italy as “Micutrin” and, in combination with betamethasone valerate, it was formulated as “Beta Micutrin” for athlete’s foot and ring worm diseases. The derivative, 3-cyanopyrroles, is more biologically active as pyrrolnitrin and very stable under light [153]. Jespers and co-workers (1993) reported a Fenpiclonil (CGA 142705) with more cytotoxicity for the representatives of *Ascomycetes, Basidiomycetes*, and *Deuteromycetes*. PRN formulated with carboxymethyl cellulose (5%) was injected intraperitoneally into mice [154] and LD_50_ was observed at a dosage of 500 mg Kg^−1^ [38]. 

In pharmacology, *in vitro* radioactive studies of pyrrolnitrin reflected that pyrrole ring is readily oxidized by enzymes undetected in urine and bile after administration [96]. Along with this, surface antigens of *Candida albicans* were released after treatment with PRN [38]. It also showed cytotoxicity at 10 µg mL^−1^ after 24 h and highest after 72 h on rat clonal pancreatic β-lines, INS-1. Thus, the compound becomes diabetogenic but appears nontoxic and insulinotropic at lower concentration [146]. PRN affected physiology of *Caenorhabditis elegans*, acted as repellent for adult nematodes to lower egg hatching by almost <50% at higher concentrations of PRN (1, 5, and 10 μg mL^−1^) after 24 h of exposure [84]. 

## 5. Conclusions

Natural bioactive PRN from different subgroups of rhizobacterial species display an array of biological properties, most prominently being the antifungal activity. Besides the leads on the formulation development and commercialization of the products for human and plant disease management, there exists tremendous scope with this small molecule for future research on making prominent functional derivatives with unmatched biological properties. The knowledge about metabolic route for biosynthesis, network of genes and enzymes linked with the intermediates, optimization of process parameters, assessment of efficient producer strains and optimized nutrient requirements of microbial species for improved PRN production need further improvement. We systematically rationalized chemistry and biological applications of PRN. However, the search for hypersecretory bacterial strains from the rhizosphere and soil habitat for economic production is being realized for maximum optimization of productivity of the molecule. Microbial systems tolerant to a wide range of organic solvents of industrial use might be a new route to economic PRN biosynthesis. Application of halogenase from high yielding bacteria could help to overcome issues of regioselectivity, dependency on chemical synthetic route and low yield of PRN. Besides, organic solvent tolerant halogenases for tailor-made synthesis and simplified downstream operations possible for PRN and green synthesis routes could also support industrial processes for PRN production.

## Figures and Tables

**Table 1:** Structural diversity of organohalogen secondary metabolites from various organisms inhabiting different habitats.

Organohalogens	Bioactivity	Halogen Type and Number	Source; Habitat	Reference(s)
**Plants**				
4-Chloroindole Ester	Plant growth promoting hormone	Cl (01)	*Pisum Sativum* (Lentil, Sweet Pea, Sea Pea, Vetch); Soil	[21,22]
3-Chloroindole acetate	Plant hormone	Cl (01)	*Ptychodero Povo Loysanica*; Marine acorn worm	[23]
Romucosine B	Plant alkaloids	Cl (01)	*Rollinia mucosa*; Tropical south America	[24]
Neoirietetrao	Diterpene	Br (01)	*Laurencia yonaguniensis*; Yonaguni island, Japan	[25]
Bromomethane	Fumigant; pesticides	Br (01)	Cabbage, Broccoli, Turnips, Rapeseeds (Family: *Brassicaceae*); Soil	[26]
2-Chloro-4-Nitrophenol	Fungicide	Cl (01)	*Stephanospora Caroticolor*; Soil	[26]
**Animals**				
Tyrosine derivative	Improving adhesion between protein fiber, sheets	Cl (01-03)	Marine Sponges, Sea fans, Gorgonians; Sea water	[27]
Diiodotyrosine	Precursor in production of thyroid hormone	I (02)	*Gorgonia Cavolii*, Sea Fan; Western Atlantic Ocean	[28]
Ecuadoran	Analgesic activity	Cl (01)	Epipedobotes; Eastern Atlantic Ocean	[29]
Tyrian Purple Dye	Dye	Br (02)	*Murex Brandaris*; Sea snail	[30]
Drosophilin A	Antibiotic	Cl (04)	*Drosophila Substrata; Ligninolytic Basidiomycetes;* overripe or rotting fruit	[31]
2,6 Dichlorophenol	Sex pheromone; growth hormone	Cl (02)	*Female; Penicillium Mold*; Decaying material	[26]
2,4 Dichlorophenol	Broad spectrum herbicides	Cl (02)	*Penicillium* Spp.; Agricultural inoculant	[26]
Epibatidine	Pain killer	Cl (01)	*Epipedobates Anthonyi* (Frog); Central; Southern cuador	[32]
**Microorganisms**				
Chloramphenicol	Antibiotic	Cl (02)	*Streptomyces venezuelae*; Soil, decaying vegetation	[33]
Chlortetracycline	Antibiotic	Cl (01)	*Streptomyces aurefaciens*; Agricultural soil	[34]
Grisiofulvin	Antifungal drug	Cl (01)	*Penicillium grisiofulvum*; Soil	[35]
Pyoluteorin	Antibiotic	Cl (02)	*Pseudomonas aeruginosa*; Rhizospheric soil	[36]
Fluoroacetic Acid	Pesticide	F (01)	*Streptomyces cattleya*; Soil	[37]
Pyrrolnitrin	Antifungal antibiotic	Cl (02)	*Burkholderia pyrrocinia, P. fluorescence, Serratia plymuthica*; Rhizospheric soil	[38]
Nucleocidin	Nucleoside antibiotic	F (01)	*Streptomyces calvus*; Soil	[39]
Vancomycin	Antibiotic	Cl (02)	*Amycolatopsis orientalis*; Soil	[40]
2′Chloropentostatin	Nucleoside antibiotic	Cl (01)	*Actinomadura* sp.; Soil	[41]
Napyradiomycin	Antibiotic	Cl (02)	*Chainia rubra*; Soil	[42]
Calicheamicin Β1	Cytotoxin	Br (01)	*Micromonospora echinospora*; Rhizospheric soil	[43]
Pyrroindomycine B	Antibiotic	Cl (01)	*Streptomyces rugosporus*; Soil	[44]
Pentabromopseudilin	Marine antibiotic	Br (05)	*Pseudomonas bromoutilis*; Coastal area	[45]
Cryptophycin A	Anticancer	Cl (01)	*Cyanobacterium*; Terrestrial, aquatic habitat	[46]
2-Chloro-4-Nitrophenol	Fungicide	Cl (01)	*Stephanospora caroticolor*; Rotting wood or plant debris	[47]
3,5 Dichloro-Hexanophenone	Inhibit fruiting body formation	Cl (02)	*Dictyostelium discoideum*; Decaying peach	[31]
Rebeccamycin	Weak Topoisomerase I Inhibitor, antitumor	Cl (02)	*Streptomyces* sp.; Rhizosphere, agricultural soil	[26]
Chlortetracycline	Antibiotic	Cl (01)	*Streptomyces aureofaciens*; Sanborn field	[48]

**Table 2:** Derivatives of pyrrolnitrin biosynthesized by *Pseudomonas aureofaciens* [56].

IUPAC Name	Common Name	Structure	Extinction Coefficient ƛ_max_ MeOH (log ε)		Molecular Formula	Molar Mass/ Molecular Weight
3-(2-amino-3-chlorophenyl)-pyrrole	Mono-chloro-amino-pyrrolnitrin (MCA)	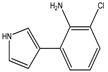	-	303 (3.52)	**C_10_H_9_ClN_2_**	**Exact Mass:** 192.05 **Mol. Wt.:** 192.64
3-chloro-4(2-amino-3-chlorophenyl)-pyrrole	Di-chloro-amino (DCA) (amino-pyrrolnitrin)	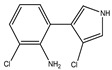	212 (4.46)	302 (3.57)	**C_10_H_8_Cl_2_N_2_**	**Exact Mass:** 226.01 **Mol. Wt.:** 227.09
2, 3 dichloro-4-(2-amino-3-chlorophenyl)-pyrrole	Tri-chloro-amino (TCA)	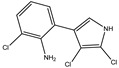	212 (4.54)	302 (3.61)	**C_10_H_7_Cl_3_N_2_**	**Exact Mass:** 259.97 **Mol. Wt.:** 261.53
3-chloro-4-(3-chloro-2nitro-phenyl)-1H pyrrole	Pyrrolnitrin (PRN)	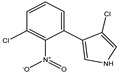	212 (4.39)	252 (3.83)	**C_10_H_6_Cl_2_N_2_O_2_**	**Exact Mass:** 255.98 **Mol. Wt.:** 257.07
2, 3 dichloro-4-(2-nitro-3-chlorophenyl) pyrrole	2-chloro-pyrrolnitrin (2-CPRN)	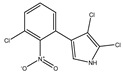	212 (4.47)	240 280	**C_10_H_5_Cl_3_N_2_O_2_**	**Exact Mass:** 289.94 **Mol. Wt.:** 291.52
2-(2-Heptenyl)-3-methyl-4(1H) quinolone	-	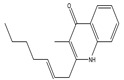	-	-	**C_17_H_21_NO**	**Exact Mass:** 255.16 **Mol. Wt.:** 255.35
2,3-dichloro-4-(2-nitrophenyl) pyrrole	Iso-pyrrolnitrin	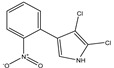	-	-	**C_10_H_6_Cl_2_N_2_O_2_**	**Exact Mass:** 255.98 **Mol. Wt.:** 257.07
3-chloro-4-(2-nitro-3-chloro-6-hydroxyphenyl) pyrrole	Oxy-pyrrolnitrin	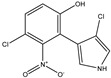	-	-	**C_10_H_6_Cl_2_N_2_O_3_**	**Exact Mass:** 271.98 **Mol. Wt.:** 273.07

**Table 3:** Characteristics of PRN production by different microbial species inhabiting several ecohabitats.

Sr. no.	Producer	Habitat	Medium	Physical Condition	Incubation Period (Days)	Concentration	Significance	Reference
1.	*Pseudomonas pyrrocinia*	-	Bouillon Medium	-	-	ND	Antibiotic, antifungal nature	[26,38]
2.	*P. aureofaciens, P. fluorescens, P. multivorans*	-	CMM, Synthetic C, E	27 °C, shaker	7	0.32–126 (µg mL^−1^)	PRN widespread in groups of *Pseudomonas*	[64]
3.	*P. aureofaciens*	-	CMM	27 °C, shaker	5	9.5 to 50 (µg mL^−1^)	Production of substituted PRN from Tryptophan analogs	[56]
4.	*P. aureofaciens*	-	CMM	30 °C, shaker	5	18.35–19.9 (µm)	Possible pathway discussed	[77]
5.	*Pseudomonas cepacia* B37w (NRRL B-14858)	Rhizosphere	Sabouraud Maltose Broth	-	6	2.133 (mg L^−1^)	Efficacy against *F. Sambucinum* incited potato dry rot disease	[59]
6.	*Pseudomonas cepacia* LT4-12- W	Apple leaves	Mineral Salt, Nutrient Broth, Kings medium B	27 °C, 200 rpm	7	1) MS: 51.50 (mg L^−1^) 2) NB: 7.20 (mg L^−1^) 3) KMB: 5.50 (mg L^−1^)	Production of phenylpyrrole metabolites with respect to time	[78]
7.	*B. cepacian*	-	Mineral Salt	27 °C, shaker	7	ND	Delays postharvest fruit rot in strawberries	[79]
8.	*Enterobacter agglomerans* IC1270	Grapes rhizosphere	Potato Dextrose Agar	Incubated on agar plate	5	ND	Possible role of a combination of Chitinases and pyrrolnitrin in antagonism	[65]
9.	*B. cepacia* NB-1	Ponds in botanical garden of Tubingen, Germany	Minimal medium	27 °C, aeration rate 0·5 vvm, stirrer speed 150 rev min^−1^, pH −7.0	5	0.54 (mg L^−1^)	PRN block ETS *Neurospora crassa* 74 A; inhibition of *Streptomycine* spp.	[66]
10.	*Burkholderia cepacia* 5.5B (ATCC 55344) Wild Type	Soil sample, North Carolina	Nutrient broth, Mineral salt	25 °C, at 200 rpm, pH 5.8	5	NB: 35.59; MS: 28.54 (mg 10^12^ cfu)	Biocontrol of *Rhizoctonia* stem rot of poinsettia	[80]
11.	*Pseudomonas fluorescens* psd	Roots of *Vigna mungo*	Standard succinate medium (SSM)	-	-	ND	Biocontrol property of plants protected from strain	[81]
12.	*Pseudomonas chlororaphis* O6	-	Nutrient broth, Mung bean medium	28 °C 200 rpm	-	1.7 (µg mL^−1^)	Regulation by glucose of PRN production influenced biocontrol of tomato leaf blight	[82]
13.	*Acinetobacter haemolyticus* A19	Wheat rhizosphere	Luria broth	-	2	15 (mg L^−1^)	Plasmid-mediated pyrrolnitrin production by *A. Haemolyticus* A19	[83]
14.	*Pseudomonas chlororaphis strain PA23*	-	M9 medium + 1 mm MgSO_4_ + 0.2% glucose	-	5	ND	Nematicidal and repellent activity against *Caenorhabditis elegans*	[84]
15.	*Serratia marcescens* ETR17	Tea rhizosphere	Semi-solid pigment producing media	30 °C	8	ND	Effective reduction of root-rot disease tea plant on talc-based formulations; Plant growth promoting activity	[85]

CMM: Citrate minimal medium; ND: not determined; NB: Nutrient broth; MS; Murashige-Skoog medium; cfu: colony forming units; KMB: King’s medium-B.

**Table 4:** Purification of pyrrolnitrin using various separation techniques with different solvent systems.

Matrix	Column	Organic Phase	Detection	Reference
Silica gel G	35 cm × 1.5 cm	Chloroform: methanol (9:1)	-	[83]
Silica gel (40 μm)	35.6 cm × 1.75 cm	Benzene: hexane (2:1); Benzene; Benzene: acetone (1:1); Acetone; methanol	TLC - bioautography	[59]
Silica gel (60 μm)	-	Chloroform: hexane (1:1, 1.5:1, 2:1, 5:1) (*v*/*v*); chloroform; chloroform-acetone (5:1, 1:1) (*v*/*v*); acetone	Bioassay with *R. solani*	[65]
Sephadex LH-20	-	Methanol	pHPLC	[66]
Silica gel 60 (0.015–0.040 mm; Merck)	-	Dichloromethane then methanol	TLC	[93]
Silica gel (H60)	-	Dichloromethane	Bioautography	[94]
Silica gel	(20 × 170 mm, Wakogel C-200)	Benzene, 10% ethyl acetatobenzene, 20% ethyl acetate benzene and finally ethyl acetate	TLC	[95]
Silicic acid	(240 × 22 mm)	Diethyl ether and methanol	-	[96]
-	RP C-18 flash	Water and methanol	TLC	[97]
-	RP C-18 (MPLC)	50% to 100% aq methanol	HPLC	[97]
Silica gel (60 μm)	-	Toluene	-	[98]

TLC: thin layer chromatography; HPLC: high performance liquid chromatography; Aq.: Aqueous; MPLC: medium pressure liquid chromatography; RP: Reverse phase; pHPLC: preparative HPLC.

**Table 5:** Several HPLC methods adopted to separate and quantify pyrrolnitrin from microbes using different solvent system.

Column	Flow Rate (mL/min^−1^)	Solvent System	Detector	Retention Time (min)	References
RP 18	2	Methanol: water (70:30; *v*/*v*)	-	-	[103]
50 mm × 4.6 mm I.D. guard	1.0	Acetonitrile: methanol: water (1:1:1)	UV (254 nm)	10	[78]
Rainin Dynamax C18 (21.4 × 250 mm)	-	Acetonitrile: water (3:2; *v*/*v*) fractions collected at 9.5 to 12.5 min and re-chromatographed on a silica column eluted with chloroform: hexane (1:1; *v/v*)	-	13.5	[79]
C-18 column, 5 µm	-	Isocratic acetonitrile: methanol: water (1:1:1)	-	-	[59]
Hypersil octyldecyl saline (2.1 mm diameter by 10 cm)	-	Water: methanol from 0%: 100 % and gradually changing up to 100%: 0%	-	between 10-15	[104]
Reverse phase 18	0.7	0 min 50% methanol in water 15 min 100 % methanol in water 17 min 100% methanol in water 20 min 50% methanol in water 25 min 50% methanol in water	UV (252 nm)	15.4	[65]
C-18 reverse phase (125 × 4.6 mm)	-	Methanol: water (70:30; *v/v*)	UV (252 nm)	-	[105]
-	1.0	2-min initialization at 10% ACN: 0.1% TFA; 20-min linear gradient to 100% ACN: 0.1% TFA	990-photodiode array detector	-	[91,106]
Nucleosil C-18		Acetonitrile: water (20 to 100%)	-	27.5	[66]
RP C-18 column	1.0	Isocratically 45% water: 30% acetonitrile: 25% methanol	-	-	[102]
C-18 RP column		10% acetonitrile: water (*v*/*v*) (both acidified with 0.1% amino acid) run for 2min linear gradient 100% acetonitrile (acidified with 0.1% amino acid)	-	18	[107]
-	-	30 ~ 60% aq acetonitrile (for 70 min)	-	68.9	[97]
Gemini C18 column (100 × 4.6 mm; 5mm particle diameter)	1.0	Isocratically 45% acetonitrile: 35% water: 20% methanol	Dionex AD20 (Dionex,Sunnyvale, CA) (225 nm)	-	[84,108]
Cosmosil C18	0.7	18 min linear gradient from 50 to 100% methanol and 0.1% trifluoracetic acid in methanol	-	-	[82]

**Table 6:** Bioactivity spectrum of pyrrolnitrin against bacteria, fungi and nematodes.

Sr. No.	Name of Test Microorganism	PRN (µg mL^−1^)	Reference
**Bacteria**			
1.	*Staphylococcus aureus* 209-P	6.2	[38]
2.	*Escherichia coli*	250	[38]
3.	*M. tuberculosis* CIP 103471	4.0	[129]
4.	*M. avium* CIP 103317	8.0	[129]
5.	*M. smegmatis* CIP 103599	16.0	[129]
6.	*M. gordonae* CIP 6427	>16.0	[129]
7.	*M. marinum* CIP 6423	>16.0	[129]
**Yeast**			
8.	*Candida albicans*	1.0	[38]
9.	*Saccharomyces cerevisiae*	10.0	[38]
10.	*Cryptococcus neoformans*	< 0.78	[136]
11.	*Candida albicans*	12.5	[137]
12.	*Cryptococcus neoformans*	0.78	[137]
13.	*Candida utilis*	10.0	[138]
**Fungi**			
14.	*Trichophyton asteroids*	0.05	[38]
15.	*Sporotrichum schenckii*	< 0.78	[136]
16.	*Penicillium atrovenetwn*	10.0	[139]
17.	*P. oxalicwn*	10.0	[139]
18.	*Sporotrichum schenckii*	3.12	[137]
19.	*Blastomyces dermatitidis*	< 0.78	[137]
20.	*Histoplasma capsulatu*	0.156	[137]
21.	*Sclerotinia sclerotiorum*	0.01	[59]
22.	*Rhizoctonia solani*	50 (µg/disc)	[97]
**Nematode**			
23.	*Caenorhabditis elegans*	0.1	[84]
